# Distinct benefit frames generate divergent effects of time scarcity mindset on prosocial behavior

**DOI:** 10.3389/fpsyg.2025.1601936

**Published:** 2025-06-02

**Authors:** Chen Yang

**Affiliations:** School of Physical Education, Qilu Normal University, Jinan, China

**Keywords:** time scarcity mindset, benefit frames, prosocial behavior, other-benefit frame, self-and-other benefit frame

## Abstract

Previous research has explored how time scarcity mindset influences prosocial behavior; however, the results have been inconsistent. The current research aimed to introduce benefit frames to examine the effect of time scarcity mindset on prosocial behavior. Inspired by the proposal that time scarcity mindset strengthens agentic (i.e., self-oriented) goals while weakening communal (i.e., other-oriented) goals, we assumed that benefit frames would moderate the impact of time scarcity mindset on prosocial behavior. We conducted a survey study (*N* = 282 participants) and an experimental study (*N* = 299 participants) to test this assumption. Our results indicated that under an others-benefit frame (i.e., benefits only to others), time scarcity mindset inhibited prosocial behavior, whereas this effect was attenuated under a self-and-other benefit frame (benefits to both oneself and others). These findings not only deepen our understanding of the effects of time scarcity mindset but also offer practical insights into how to mitigate its detrimental effect on prosocial behavior.

## 1 Introduction

Time scarcity is defined as the personal feeling of having too many things to do and not enough time to accomplish them (Szollos, 2009), and it has become one of the most ubiquitous experiences in daily life worldwide. For example, in the United States, a Gallup poll reported that 80% of Americans experienced time scarcity (Giurge et al., 2020). Similar trends have been observed in Japan (Urakawa et al., 2020), Australia (Pocock et al., 2001), Canada (Spinney and Millward, 2010), and China (Li et al., 2015). This lack of sufficient time is not merely an occasional inconvenience associated with specific tasks; instead, it has become a chronic aspect of many people’s everyday lives. Notably, prosocial behavior is a key marker of healthy social functioning (Dovidio et al., 2006). This raises the question of whether feeling chronically time-scarce might undermine individuals’ prosocial engagement. Accordingly, emerging research has begun to examine how time scarcity mindset affects prosocial behavior, given that time is often treated as a priced resource (Pfeffer and DeVoe, 2012) and that prosocial behavior inevitably requires individuals to invest their time and money (Reed II et al., 2016).

However, prior research examining the effect of time scarcity mindset on prosocial behavior has yielded paradoxical results. Some studies have found that time scarcity mindset makes individuals more self-interested, thereby reducing purely altruistic prosocial behavior (Capraro and Cococcioni, 2016; Krawczyk and Sylwestrzak, 2018; Teoh et al., 2020). In contrast, other studies have found that time scarcity can actually improve cooperative prosocial behavior that benefits both oneself and others (Bouwmeester et al., 2017; Rand, 2016; Rand et al., 2012). We propose that these conflicting findings stem from a critical difference in the prosocial context—specifically, whether the helper also benefits from helping the recipient. Research has shown that prosocial behavior can be driven by either self-interested or other-oriented goals (Wang et al., 2021). Moreover, time scarcity mindset tends to activate self-interested goal orientations while diminishing other-oriented ones (Jiang et al., 2024). Therefore, to reconcile the contradictory findings from previous research, the current study examined whether the benefit frame of a prosocial context (i.e., whether prosocial behavior also benefits the helper) moderates the impact of time scarcity mindset on prosocial behavior.

### 1.1 Paradoxical effects of time scarcity mindset on prosocial behavior

Time scarcity mindset is typically defined as the personal feeling of having too many things to do but not enough time to accomplish them (Szollos, 2009). This mindset is not merely a chronic, overarching perception; rather, it can arise in specific situations or tasks (Jiang et al., 2024). In other words, time scarcity mindset is relatively flexible and can fluctuate over time. More importantly, time scarcity mindset has been conceptualized as a motivational orientation that heightens individuals’ focus on personal goal attainment, often leading to the deprioritization of others’ needs or concerns in decision-making contexts (Jiang et al., 2024).

Prosocial behavior refers to actions that a significant segment of society or one’s social group considers generally beneficial to other people (Penner et al., 2005). This behavior is vital for facilitating smooth social interactions and strengthening group solidarity (Fetchenhauer et al., 2006; Lawler et al., 2009). As a positive form of social action, prosocial behavior often involves generosity, cooperation, and charitable giving (Fehr et al., 2002; Wispé, 1972). Researchers have identified two distinct types of motives underlying prosocial behavior (Wang et al., 2021). On the one hand, prosocial actions may be driven by self-serving or egoistic motives (Batson, 2011; Keltner et al., 2014). On the other hand, prosocial behavior can stem from selfless, purely altruistic motives (Batson and Powell, 2003).

Previous findings on the effect of time scarcity mindset on prosocial behavior have been inconsistent. On the one hand, some studies have suggested that this mindset inhibits prosocial behavior. For example, when individuals feel short on time, they tend to focus on self-relevant information (Teoh et al., 2020) and prioritize self-serving responses (Capraro and Cococcioni, 2016). Consequently, they exhibit less generosity and other unselfish behaviors (Krawczyk and Sylwestrzak, 2018), which ultimately impede charitable giving (Mrkva, 2017). In contrast, other studies have found that time scarcity can actually increase prosocial behavior (Rand, 2016). For instance, Rand et al. (2012) reported that time pressure led to greater prosociality, a finding corroborated by Bouwmeester et al. (2017), who observed that time pressure increased cooperation.

We propose that the differences in how prosocial behavior is framed could explain the seemingly divergent effect of time scarcity mindset on prosocial behavior. Specifically, Teoh et al. (2020) conceptualized prosocial behavior as a purely altruistic act, Krawczyk and Sylwestrzak (2018) emphasized its non-selfish nature, and Mrkva (2017) viewed it as a charitable choice. In contrast, Rand et al. (2012) and Bouwmeester et al. (2017) framed prosocial behavior in terms of cooperation that aligns with “rational” self-interest. These differing conceptualizations closely correspond to the two primary motives for prosocial behavior—one that is selfless and the other that is self-serving. Moreover, Teoh and Hutcherson (2022) demonstrated that distinct prosocial contexts—namely, pure altruism and cooperation—affect individuals’ prosocial choices under *objective time constraints*. Therefore, the present research examined prosocial behavior under these two distinct benefit frames to clarify how a *subjective time scarcity mindset* influences prosocial behavior.

### 1.2 Moderating effect of the benefit frame on prosocial behavior

Given that prosocial behavior, in terms of motivation, can be framed as benefiting either solely others or both oneself and others (Wang et al., 2021), the benefit frame refers to how the distribution of outcomes in prosocial contexts is perceived (Teoh and Hutcherson, 2022). Specifically, the benefit frame can be categorized into two key types: the self-and-other benefit frame and the other-benefit frame (Teoh and Hutcherson, 2022). The self-and-other benefit frame emphasizes prosocial behavior driven not only by altruistic intentions but also by self-interested motives, such as enhancing personal reputation (Song et al., 2024; Wang et al., 2021), gaining monetary rewards, or avoiding punishments (Fehr and Fischbacher, 2004). In contrast, the other-benefit frame focuses purely on altruistic prosocial behavior in which individuals act exclusively to benefit others without personal gains (Guo et al., 2022; Wang et al., 2021).

We argue that the benefit frame moderates the impact of time scarcity mindset on prosocial behavior in two ways. First, compared to the self-and-other benefit frame, the other-benefit frame lacks self-relevant information that individuals experiencing time scarcity tend to prioritize. Specifically, time scarcity mindset heightens individuals’ focus on self-relevant cues, guiding their behavioral choices accordingly (Karau and Kelly, 1992). Therefore, when prosocial behavior is framed as benefiting only others (i.e., lacking self-relevant incentives), individuals experiencing high levels of time scarcity are likely to exhibit decreased prosocial engagement (Teoh et al., 2020).

Second, the other-benefit frame fails to satisfy the self-interested motivations amplified by time scarcity mindset. When prosocial behaviors are framed solely in terms of benefiting others, engaging in these behaviors addresses altruistic goals but does not fulfill self-centered motives (Wang et al., 2021). As time scarcity strengthens self-interested motives and weakens the inclination to prioritize others’ welfare (Jiang et al., 2024), individuals experiencing time scarcity perceive purely altruistic prosocial behavior as misaligned with their immediate goals. Consequently, these individuals are less inclined to engage in prosocial behavior. Conversely, under the self-and-other benefit frame, individuals experiencing time scarcity can simultaneously achieve altruistic intentions and satisfy their self-interested motives, resulting in prosocial engagement comparable to that of individuals not experiencing time scarcity.

Based on the foregoing theoretical reasoning and empirical findings, we proposed the following hypothesis: *the benefit frame moderates the impact of time scarcity mindset on prosocial behavior*. Specifically, time scarcity mindset is more likely to reduce prosocial behavior when it is framed as solely benefiting others, whereas this inhibitory effect is attenuated when prosocial behavior benefits both oneself and others.

### 1.3 Overview of the current research

To test our hypothesis, we conducted two studies. Study 1 used a cross-sectional survey design to investigate the correlations between time scarcity mindset, benefit frame, and prosocial intentions. Study 2 employed an experimental design to directly manipulate both time scarcity mindset and the benefit frame. Furthermore, Study 2 measured actual prosocial behavior, thereby extending the findings of Study 1 from intentions to observable behavioral outcomes.

All data and stimulus materials are available on the Open Science Framework.^1^

## 2 Study 1

To initially test the hypothesis, Study 1 assessed participants’ self-reported scores on the variables of interest. We predicted that participants’ time scarcity mindset would significantly negatively predict prosocial behavior under the other-benefit frame. However, under the self-and-other benefit frame, we anticipated that this negative effect would diminish or even reverse.

### 2.1 Methods

#### 2.1.1 Participants

Given that this study primarily focuses on the relationship between time scarcity and prosocial behavior under different benefit frames and that research findings remain robust when the sample size reaches 250 or more (Da Silva Frost and Ledgerwood, 2020), we have established 250 as the minimum sample size. A total of 282 Chinese participants were recruited online via Credamo^2^ (112 men; *M*_age_ = 29.34 years, *SD*_age_ = 7.13 years, range = 19–57 years). Of these, 27.7% were undergraduate students, 67.7% were employed full-time, and 4.6% were self-employed.

#### 2.1.2 Procedure and materials

After providing informed consent, the participants anonymously completed the following measures. The presentation order of the two prosocial behavior scales (other-benefit and self-and-other benefit frames) was counterbalanced across participants to control for potential order effects.

**Time scarcity mindset.** To assess participants’ time scarcity mindset, they first completed an 8-item scale adapted from previous research (Kasser and Sheldon, 2009; e.g., “My life has been too rushed,” 1 = *completely disagree*, 7 = *completely agree*). The scores on all items were averaged to create an indicator of time scarcity mindset (*α* = 0.83). Higher scores indicated a stronger time scarcity mindset.

**Prosocial behavior under the other-benefit frame.** To measure prosocial behavior under the other-benefit frame, the participants completed a 15-item scale adapted from previous research (Caprara et al., 2005; e.g., “Even if there is no personal benefit for me, I am pleased to help my friends or colleagues in their activities.”; 1 = *completely disagree*, 7 = *completely agree*). Scores on all items were averaged to create an indicator of prosocial behavior under the other-benefit frame (*α* = 0.96). Higher scores indicated a strong willingness to engage in prosocial behavior under the other-benefit frame.

**Prosocial behavior under the self-and-other benefit frame.** Similarly, to measure prosocial behavior under the self-and-other benefit frame, the participants completed a 15-item scale adapted from previous research (Caprara et al., 2005; e.g., “If there are personal benefits for me, I am pleased to help my friends or colleagues in their activities.”; 1 = *completely disagree*, 7 = *completely agree*). Scores on all items were averaged to create an indicator of prosocial behavior under the self-and-other benefit frame (*α* = 0.94). Higher scores indicated a strong willingness to engage in prosocial behavior under the self-and-other benefit frame.

**Control variables.** The participants reported their objective monthly income and completed an 8-item scale measuring money scarcity mindset, which is in line with previous research investigating the effect of time scarcity mindset (Kasser and Sheldon, 2009).

### 2.2 Results

To preliminarily examine the relationships between time scarcity mindset and prosocial behavior under two distinct benefit frames, descriptive and correlation analyses were conducted using SPSS 20.0. The means and standard deviations for each variable and the correlations between all variables are presented in Table 1. The results demonstrated that time scarcity mindset was negatively associated with prosocial behavior under the other-benefit frame (*p* = 0.001) but was positively associated with prosocial behavior under the self-and-other benefit frame (*p* = 0.006). In addition, a direct comparison using Fisher’s r-to-z transformation revealed that the difference between these two correlations was statistically significant (*z* = −4.30, *p* < 0.001).

**Table 1:** The mean, standard deviation, and intercorrelations of the variables in Study 1.

Variables	*M* (*SD*)	1	2	3	4	5	6
1. Time scarcity mindset	3.95 (1.14)						
2. Prosocial behavior under the other-benefit frame	5.07 (1.16)	−0.20^**^					
3. Prosocial behavior under the self-and-other benefit frame	5.45 (0.91)	0.16^**^	−0.04				
4. Money scarcity mindset	3.71 (0.44)	−0.02	0.17^**^	−0.06			
5. Personal monthly income	4.26 (1.89)	−0.24^***^	0.07	−0.02	−0.02		
6. Age	29.34 (7.13)	−0.20^**^	0.08	−0.14^*^	−0.05	0.62^***^	
7. Sex	–	0.02	−0.06	−0.02	0.06	−0.08	−0.14^*^

^***^p < 0.001, ^**^p < 0.01, ^*^p < 0.05. Sex: male = 1, female = 2.

To robustly test the hypothesized relationships between time scarcity mindset and prosocial behavior under two distinct benefit frames, path analysis was conducted using Mplus 7.0. The results indicated a strong fit with the data: *χ*^2^ (0, 282) = 0.000, *p* < 0.001, RMSEA = 0.000, CFI = 1.000, TLI = 1.000, and SRMR = 0.000. The analysis revealed that the relationship between time scarcity mindset and prosocial behavior under the other-benefit frame was significantly negative (*B* = −0.185, *SE* = 0.05, *β* = −0.215, *p* < 0.001), whereas the relationship between time scarcity mindset and prosocial behavior under the self-and-other benefit frame was significantly positive (*B* = 0.109, *SE* = 0.04, *β* = 0.163, *p* = 0.007). These relationships remained significant even after controlling for monthly income (other-benefit frame: *B* = −0.025, *SE* = 0.05, *β* = −0.042, *p* = 0.617; self-and-other benefit frame: *B* = −0.013, *SE* = 0.04, *β* = −0.028, *p* = 0.746), education level (other-benefit frame: *B* = −0.121, *SE* = 0.07, *β* = −0.145, *p* = 0.073; self-and-other benefit frame: *B* = 0.024, *SE* = 0.05, *β* = 0.037, *p* = 0.653), sex (other-benefit frame: *B* = −0.068, *SE* = 0.09, *β* = −0.058, *p* = 0.472; self-and-other benefit frame: *B* = 0.052, *SE* = 0.08, *β* = 0.057, *p* = 0.492), age (other-benefit frame: *B* = 0.083, *SE* = 0.07, *β* = 0.092, *p* = 0.261; self-and-other benefit frame: *B* = −0.009, *SE* = 0.06, *β* = −0.012, *p* = 0.883), and money scarcity mindset (other-benefit frame: *B* = 0.019, *SE* = 0.20, *β* = 0.007, *p* = 0.926; self-and-other benefit frame: *B* = −0.322, *SE* = 0.16, *β* = −0.165, *p* = 0.044). The results showed a good fit with the data: *χ*^2^ (0, 282) = 0.000, *p* < 0.001, RMSEA = 0.000, CFI = 1.000, TLI = 1.000, and SRMR = 0.000. Specifically, the relationship between time scarcity mindset and prosocial behavior under the other-benefit frame remained significantly negative (*B* = −0.222, *SE* = 0.07, *β* = −0.275, *p* = 0.001), whereas the relationship between time scarcity mindset and prosocial behavior under the self-and-other benefit frame remained significantly positive (*B* = 0.128, *SE* = 0.05, *β* = 0.202, *p* = 0.017).

## 3 Study 2

Study 2 served a twofold purpose. First, it aimed to offer causal evidence for the results found in Study 1. In Study 2, time scarcity mindset was manipulated by assigning the participants to either a high or low time scarcity condition. Second, Study 2 sought to extend the findings of Study 1 to the behavioral level. To this end, real monetary incentives were offered to the participants for engaging in prosocial behavior.

### 3.1 Methods

#### 3.1.1 Participants

Based on a small-to-medium effect size of interest (Cohen, 1992) of *f* = 0.17 (da Silva Frost and Ledgerwood, 2020), a sample size of 274 was determined to be sufficient to achieve 80% power at *α* = 0.05 (two-tailed) for detecting the moderating effect of the benefit frame on the relationship between time scarcity mindset and prosocial behavior. Ultimately, 300 Chinese participants were recruited online via Credamo (see text footnote 2). One participant was excluded after failing the attention checks, resulting in a final sample size of 299 participants (104 men; *M*_age_ = 31.71 years, *SD*_age_ = 8.05 years, range = 20–58). These participants were randomly and evenly assigned to one of the four conditions (*χ*^2^ (1, 299) = 0.003, *p* = 0.954) in a 2 (time scarcity mindset: high, low) × 2 (benefit frame: other, self-and-other) between-subjects factorial design. The conditions were as follows: the high time-scarcity mindset and other-benefit frame condition (*n* = 74), high time scarcity mindset and self-and-other benefit frame condition (*n* = 75), low time scarcity mindset and other-benefit frame condition (*n* = 75), and low time scarcity mindset and self-and-other benefit condition (*n* = 75). The participants were compensated with a baseline payment of 1 CNY (approximately US $0.14) and an extra payment between 0 and 0.75 CNY (approximately US $0.10) based on their prosocial decisions in the study.

#### 3.1.2 Procedure and materials

After providing informed consent, the participants were presented with the following scenario: “Imagine that you have been working at a company called Hawei for five years and you reside in a community named Chunhui with your family of three.”

**Time scarcity mindset manipulation.** Time scarcity mindset was experimentally manipulated using an imaginary scenario adapted from Yuan and Sun (2024). The participants assigned to the high time scarcity mindset condition received a description indicating that they were overwhelmed with numerous tasks, both at work and home, leading to a perception of high time scarcity. Conversely, those assigned to the low time scarcity mindset condition were instructed to imagine having sufficient time to complete all work and home tasks, thereby fostering a low perception of time scarcity. Further details of the scenarios are available in Supplementary material.

**Benefit frame manipulation.** The benefit frame was manipulated using an adapted version of the dictator game (Ensminger and Cook, 2014). The participants were randomly assigned to either the other-benefit frame condition or the self-and-other benefit frame condition. In the other-benefit frame condition, the participants were informed that their donations (X tokens) would remain entirely anonymous and provide no personal benefit (economic or social). The extra monetary incentive for this condition was calculated as (100 – X) tokens, at a rate of 1 CNY per 200 tokens. In the self-and-other benefit frame condition, the participants were informed that their donations, while anonymous, could also result in personal benefits, such as invitations to community charity events offering networking opportunities, skill development, career advancement, and educational resources for children. The incentive structure included both altruistic and personal gains, calculated as (100 + 0.5X) tokens at a rate of 1 CNY per 200 tokens.

**Manipulation check for time scarcity mindset.** To verify the success of the time scarcity mindset manipulation, the participants then completed three items (Yuan and Sun, 2024): “Living in the imagined scenario above, I feel pressed for time,” “I am always running out of time,” and “I am often in a hurry.” Each item was rated using a scale from 1 = strongly disagree to 7 = strongly agree. Scores on these items were averaged to establish an indicator of time scarcity mindset (*α* = 0.98). Higher scores suggested a stronger perception of time scarcity.

**Manipulation check for the benefit frame.** To verify the success of the manipulation of the benefit frame, the participants then completed three items adapted from Study 1: “Living in the imagined scenario above, donating is beneficial to me personally,” “Donating not only helps others but also benefits me,” and “Donating is solely to help others and has no personal benefit for me (Reverse-coded).” Each item was rated using a scale from 1 = strongly disagree to 7 = strongly agree. Scores on these items were averaged to establish an indicator of the benefit frame (*α* = 0.97). Higher scores indicated a stronger perception that prosocial behavior provided benefits to both the self and others.

**Prosocial behavior.** The participants’ prosocial behavior was measured through a charitable donation task. Each participant received 1,000,000 tokens, from which they could voluntarily donate to a community fundraising initiative aimed at assisting disadvantaged families. The participants made anonymous, one-time donations using a slider scale ranging from 0 to 100 in units of 10,000 tokens. The total amount donated served as the behavioral measure of prosociality, with higher donations indicating greater prosocial behavior.

### 3.2 Results

#### 3.2.1 Manipulation check for time scarcity mindset

We conducted a 2 (time scarcity mindset: high, low) × 2 (benefit frame: other, self-and-other) between-subjects ANOVA on perceived time scarcity. The main effect of time scarcity mindset was significant, with *F* (1, 295) = 8601.86, *p* < 0.001, *η*_p_^2^ = 0.967, and 90% CI [0.962, 0.971]. The participants in the high time scarcity mindset condition (*M* = 6.52, *SD* = 0.50) reported a significantly higher perceived time scarcity than those in the low time scarcity condition (*M* = 1.41, *SD* = 0.45). Neither the main effect of the benefit frame, with *F* (1, 295) = 0.072, *p* = 0.789, *η*_p_^2^ = 0.000, and 90% CI [0.000, 0. 010], nor the interaction between time scarcity mindset and the benefit frame, with *F* (1, 295) = 0.566, *p* = 0.452, *η*_p_^2^ = 0.002, and 90% CI [0.000, 0.019], was significant. Thus, the time scarcity mindset manipulation was successful.

#### 3.2.2 Manipulation check for the benefit frame

We conducted a 2 (time scarcity mindset: high, low) × 2 (benefit frame: other, self-and-other) between-subjects ANOVA on the perception that prosocial behavior provides benefits to both the self and others. The main effect of the benefit frame was significant, with *F* (1, 295) = 485.97, *p* < 0.001, *η*_p_^2^ = 0.622, and 90% CI [0.569, 0.665]. The participants in the self-and-other benefit condition (*M* = 5.80, *SD* = 0.90) perceived prosocial behavior as providing greater benefits to both the self and others than those in the other-benefit condition (*M* = 2.57, *SD* = 1.54). Neither the main effect of time scarcity mindset, with *F* (1, 295) = 0.22, *p* = 0.643, *η*_p_^2^ = 0.001, and 90% CI [0.000, 0.014], nor the interaction, with *F* (1, 295) = 0.379, *p* = 0.539, *η*_p_^2^ = 0.001, and 90% CI [0.000, 0.017], was significant. Thus, the benefit frame manipulation was successful.

#### 3.2.3 Moderating effect of the benefit frame

To test the moderating impact of the benefit frame, we conducted a 2 (time scarcity mindset: high, low) × 2 (benefit frame: other, self-and-other) between-subjects ANOVA on the prosocial behavior measure. The main effect of time scarcity mindset was significant, with *F* (1, 295) = 8.74, *p* = 0.003, *η*_p_^2^ = 0.029, and 90% CI [0.006, 0.067]. Participants in the high time scarcity mindset condition exhibited significantly less prosocial behavior (*M* = 53.07, *SD* = 36.66) compared to those in the low time scarcity mindset condition (*M* = 64.37, *SD* = 31.80). In addition, the main effect of the benefit frame was significant, with *F* (1, 295) = 13.22, *p* < 0.001, *η*_p_^2^ = 0.043, and 90% CI [0.013, 0.086]. Participants in the other-benefit condition engaged in less prosocial behavior (*M* = 51.76, *SD* = 35.06) than those in the self-and-other benefit condition (*M* = 65.68, *SD* = 33.05).

**Figure 1:**
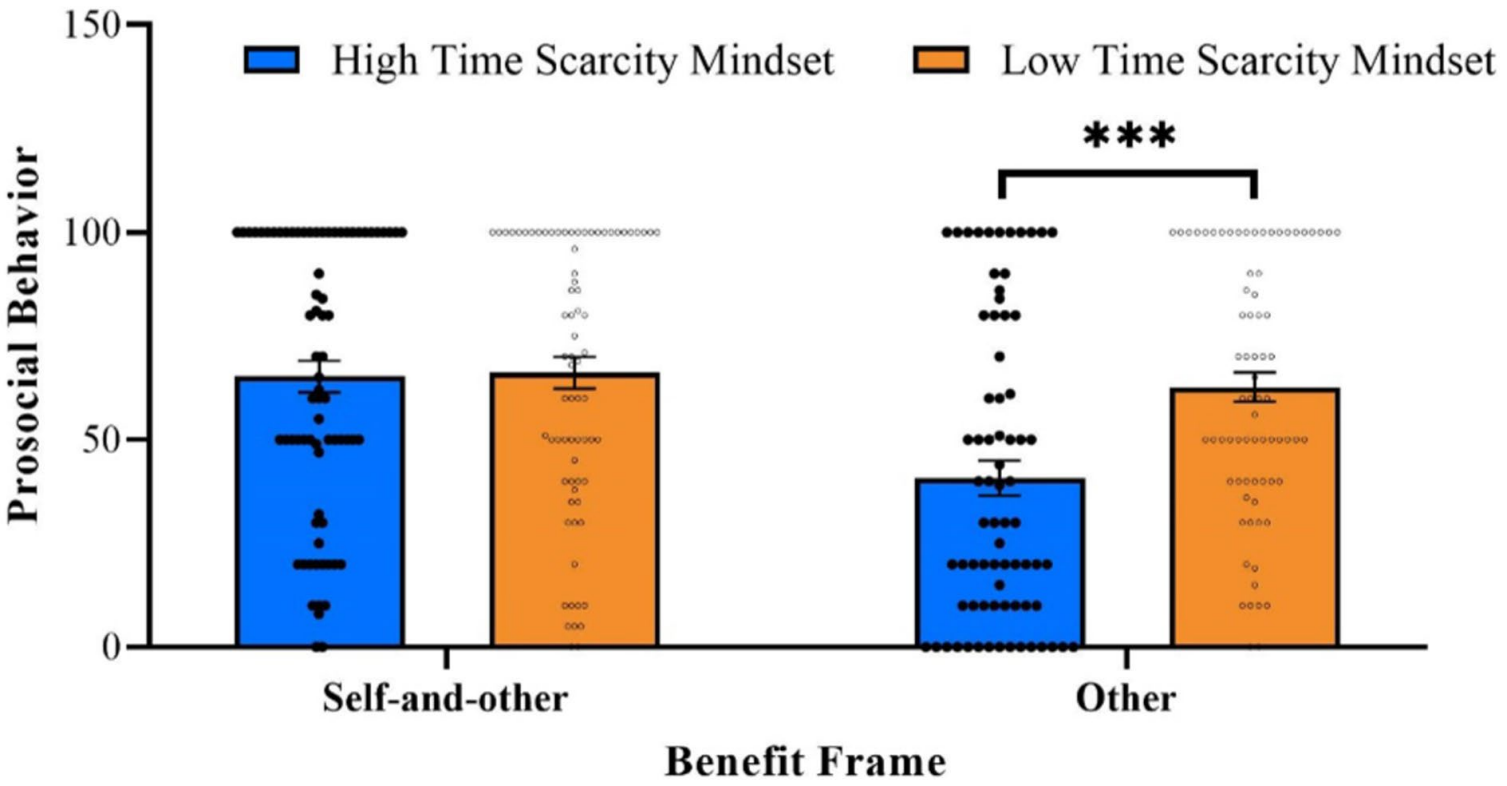
Prosocial behavior as a function of time scarcity mindset and the benefit frame in Study 2. Error bars represent the standard error of the mean. Dots depict jittered individual data points.

As predicted, the interaction effect was significant (see Figure 1), with *F* (1, 295) = 7.44, *p* = 0.007, *η*_p_^2^ = 0.025, and 90% CI [0.004, 0.061]. Simple effects analysis revealed that under the other-benefit condition, participants with high time scarcity mindset (*M* = 40.74, *SD* = 35.95) engaged in significantly less prosocial behavior than those with low time scarcity mindset (*M* = 62.63, *SD* = 30.70), with *F* (1, 295) = 16.10, *p* < 0.001, *η*_p_^2^ = 0.052, and 90% CI [0.018, 0.098]. In contrast, under the self-and-other benefit condition, prosocial behavior did not significantly differ between the high (*M* = 65.24, *SD* = 33.34) and low (*M* = 66.12, *SD* = 32.98) time scarcity mindset conditions, with *F* (1, 295) = 0.03, *p* = 0.871, *η*_p_^2^ = 0.000, and 90% CI [0.000, 0.004].

Study 2 provided causal evidence supporting our hypothesis that the benefit frame moderates the effect of time scarcity mindset on prosocial behavior. Specifically, time scarcity mindset significantly reduced prosocial behavior when it was framed as benefiting solely others; however, this inhibiting effect was eliminated when prosocial behavior was framed as benefiting both the self and others.

## 4 General discussion

Across two studies, we examined whether the benefit frame moderates the impact of time scarcity mindset on prosocial behavior. Our findings supported the hypothesis that distinct benefit frames generate divergent effects of time scarcity mindset on prosocial behavior. Specifically, when prosocial behavior was framed solely as benefiting others, time scarcity mindset significantly decreased prosocial intentions (Study 1) and reduced prosocial behavior (Study 2). However, when prosocial behavior was framed as benefiting both the self and others, the negative impact of time scarcity mindset on prosocial behavior was eliminated.

The current research helps to figure out the paradoxical findings from prior studies regarding the impact of time scarcity mindset on prosocial behavior. Although some studies have suggested that time scarcity mindset reduces prosocial behavior (Capraro and Cococcioni, 2016; Krawczyk and Sylwestrzak, 2018; Teoh et al., 2020), others have indicated the opposite effect (Bouwmeester et al., 2017; Rand, 2016; Rand et al., 2012). Based on the distinct motives of prosocial behavior, the current research integrated these seemingly conflicting results by introducing the benefit frame of prosocial behavior. Our results revealed that time scarcity mindset reduced prosocial behavior only when it was framed as benefiting others. This is because purely altruistic behavior does not align with the self-interested motivations heightened by time scarcity (Jiang et al., 2024). However, the effect of time scarcity mindset on prosocial behavior disappeared when prosocial behavior was framed as benefiting both the self and others, as this framing satisfies the need of individuals with time scarcity mindset to prioritize personal interests and goals (Jiang et al., 2024). It is a reminder to scholars that, beyond merely focusing on the level of prosocial behavior, the benefit frame of prosocial behavior should also be considered when examining the effect of time scarcity mindset on prosocial behavior.

The current research contributes to the literature by clarifying the divergent effects of time scarcity mindset on prosocial behavior. While previous studies have primarily examined how objective time pressure influences prosocial decision-making (Teoh and Hutcherson, 2022), our findings extend this line of inquiry by focusing on subjective perceptions of time scarcity. Specifically, Teoh and Hutcherson (2022) demonstrated that distinct prosocial contexts—namely, pure altruism versus cooperation—shape individuals’ information search strategies and, in turn, affect their prosocial choices under time constraints. Given that time scarcity mindset is typically conceptualized as the subjective experience of objective time pressure (Szollos, 2009), the current research provides novel evidence that its impact on prosocial behavior is moderated by the benefit framing of the context (i.e., other-benefit vs. self-and-other benefit). These findings suggest a convergence between objective and subjective perspectives: the framing of prosocial contexts not only influences behavior under actual environmental constraints but also shapes the cognitive interpretation of those constraints, thereby guiding prosocial decision-making.

The current research makes a significant contribution to the literature on the consequences of time scarcity mindset. Previous research has mainly focused on the intrapersonal consequences of time scarcity mindset, such as increased physical health issues (Zuzanek, 2004), psychological distress (Roxburgh, 2004), and impaired cognitive performance (Shah et al., 2012; Young et al., 2012). Emerging research has also begun to explore the interpersonal consequences of time scarcity mindset, such as objectification (Jiang et al., 2024). Building on this literature, our research identifies a crucial boundary condition—the benefit frame of prosocial behavior—that shapes the interpersonal consequences of time scarcity. By revealing that benefit frames align with fundamental agentic (self-oriented; Abele and Wojciszke, 2014) and communal (other-oriented; Abele and Wojciszke, 2014) goal orientations, our research provides evidence that time scarcity mindset distinctly influences these orientations. Future studies could further explore interpersonal behaviors involving trade-offs between agentic and communal goals under varying degrees of time scarcity.

The findings from the current research have practical implications for encouraging prosocial behavior, particularly in time-scarcity contexts. In modern society, time scarcity is becoming increasingly prevalent (Jiang et al., 2024; Zhao et al., 2024). Given the increasing prevalence of time scarcity in modern societies (Jiang et al., 2024; Zhao et al., 2024), organizations and policymakers could strategically frame prosocial activities to emphasize mutual benefits. For instance, companies seeking to foster a prosocial organizational culture may highlight personal benefits, such as enhanced reputation, expanded professional networks, or increased psychological wellbeing. Similarly, charitable organizations could frame donation appeals to underscore both beneficiary outcomes and donor benefits, including personal fulfillment or social recognition. At the policy level, governments and social institutions can employ framing strategies that highlight mutual gains from prosocial actions, thereby sustaining engagement and cultivating a more altruistic and socially cohesive society.

The current research also has some limitations that should be addressed in future studies. First, the current research examined the interactive effects of the benefit frame and time scarcity mindset on prosocial behavior; future research could explore the psychological mechanism underlying these interactive effects. Second, the sample size of Study 1 (*N* < 300) was relatively small. Despite this limitation, Study 2 utilized an experimental design with random assignment, which provided stronger causal evidence for the observed effects, effectively complementing the correlational findings of Study 1.

## Data Availability

The raw data supporting the conclusions of this article will be made available by the authors without undue reservation.

## Footnotes

^1^ https://osf.io/ezmxu/?view_only=5c789099adec44d49f1452d93ded7626

^2^ https://www.credamo.com/

## References

[ref1] Abele A. E. Wojciszke B. (2014). Communal and agentic content in social cognition: a dual perspective model. Adv. Exp. Soc. Psychol. 50, 195–255. doi: 10.1016/B978-0-12-800284-1.00004-7, PMID: 40394332

[ref2] Batson C. D. (2011). Altruism in humans. New York, NY: Oxford University Press.

[ref3] Batson C. D. Powell A. A. (2003). “Altruism and prosocial behavior,” in Handbook of psychology. ed. Weiner I. B.. Hoboken, NJ: Wiley. 463–484.

[ref4] Bouwmeester S. Verkoeijen P. Aczel B. Barbosa F. Bègue L. Brañas-Garza P.. (2017). Registered replication report: Rand, Greene, and Nowak (2012). Perspect. Psychol. Sci. 12, 527–542. doi: 10.1177/1745691617693624, PMID: 28475467 PMC5453400

[ref5] Caprara G. V. Steca P. Zelli A. Capanna C. (2005). A new scale for measuring adults’ prosocialness. Eur. J. Psychol. Assess. 21, 77–89. doi: 10.1027/1015-5759.21.2.77

[ref6] Capraro V. Cococcioni G. (2016). Rethinking spontaneous giving: extreme time pressure and ego-depletion favor self-regarding reactions. Sci. Rep. 6:27219. doi: 10.1038/srep27219, PMID: 27251762 PMC4890119

[ref7] Cohen J. (1992). A power primer. Psychol. Bull. 112, 155–159. doi: 10.1037/0033-2909.112.1.155, PMID: 19565683

[ref8] Da Silva Frost A. Ledgerwood A. (2020). Calibrate your confidence in research findings: a tutorial on improving research methods and practices. J. Pac. Rim Psychol. 14:E14. doi: 10.1017/prp.2020.7

[ref9] Dovidio J. F. Piliavin J. A. Schroeder D. A. Penner L. A. (2006). The social psychology of prosocial behavior. 1st Edn. Mahwah, NJ: Psychology Press. 10.1146/annurev.psych.56.091103.070141 15709940

[ref10] Ensminger J. Cook K. (2014). “Prosociality in rural America: evidence from dictator, ultimatum, public goods, and trust games” in Foundations of human sociality: economic experiments and ethnographic evidence from fifteen small-scale societies (New York: Russell Sage Foundation), 194–231.

[ref11] Fehr E. Fischbacher U. (2004). Social norms and human cooperation. Trends Cogn. Sci. 8, 185–190. doi: 10.1016/j.tics.2004.02.007 15050515

[ref12] Fehr E. Fischbacher U. Gächter S. (2002). Strong reciprocity, human cooperation, and the enforcement of social norms. Hum. Nat. 13, 1–25. doi: 10.1007/s12110-002-1012-7, PMID: 26192593

[ref13] Fetchenhauer D. Buunk B. Flache A. Buunk A. P. Lindenberg S. (2006). Solidarity and prosocial behavior: an integration of sociological and psychological perspectives. New York, NY: Springer Science and Business Media.

[ref14] Giurge L. M. Whillans A. V. West C. (2020). Why time poverty matters for individuals, organisations and nations. Nat. Hum. Behav. 4, 993–1003. doi: 10.1038/s41562-020-0920-z, PMID: 32747805

[ref15] Guo Y. Guo Q. Liu Z. Liu H. (2022). Moral identity and empathy promote prosocial behavior only toward blameless AIDS patients. Scand. J. Psychol. 63, 229–237. doi: 10.1111/sjop.12786, PMID: 34734420

[ref16] Jiang X. Zhang N. Sun X. Liu Z. Wang Y. L. (2024). Being pressed for time leads to treating others as things: exploring the relationships among time scarcity, agentic and communal orientation and objectification. Br. J. Soc. Psychol. 63, 1318–1338. doi: 10.1111/bjso.12729, PMID: 38317579

[ref17] Karau S. J. Kelly J. R. (1992). The effects of time scarcity and time abundance on group performance quality and interaction process. J. Exp. Soc. Psychol. 28, 542–571. doi: 10.1016/0022-1031(92)90045-L

[ref18] Kasser T. Sheldon K. M. (2009). Time affluence as a path toward personal happiness and ethical business practice: empirical evidence from four studies. J. Bus. Ethics 84, 243–255. doi: 10.1007/s10551-008-9696-1

[ref19] Keltner D. Kogan A. Piff P. K. Saturn S. R. (2014). The sociocultural appraisals, values, and emotions (SAVE) framework of prosociality: core processes from gene to meme. Annu. Rev. Psychol. 65, 425–460. doi: 10.1146/annurev-psych-010213-115054, PMID: 24405363

[ref20] Krawczyk M. Sylwestrzak M. (2018). Exploring the role of deliberation time in non-selfish behavior: the double response method. J. Behav. Exp. Econ. 72, 121–134. doi: 10.1016/j.socec.2017.12.004

[ref21] Lawler E. J. Thye S. R. Yoon J. (2009). Social commitments in a depersonalized world. New York, NY: Russell Sage Foundation.

[ref22] Li A. Yan L. Wang X. Ma X. Li F. (2015). The double-edged effect and mechanism of time pressure. Adv. Psychol. Sci. 23, 1627–1636. doi: 10.3724/sp.j.1042.2015.01627

[ref23] Mrkva K. (2017). Giving, fast and slow: reflection increases costly (but not uncostly) charitable giving. J. Behav. Decis. Mak. 30, 1052–1065. doi: 10.1002/bdm.2023

[ref24] Penner L. A. Dovidio J. F. Piliavin J. A. Schroeder D. A. (2005). Prosocial behavior: multilevel perspectives. Annu. Rev. Psychol. 56, 365–392. doi: 10.1146/annurev.psych.56.091103.070141, PMID: 15709940

[ref25] Pfeffer J. DeVoe S. E. (2012). The economic evaluation of time: organizational causes and individual consequences. Res. Organ. Behav. 32, 47–62. doi: 10.1016/j.riob.2012.11.001

[ref26] Pocock S. J. McCormack V. Gueyffier F. Boutitie F. Fagard R. H. Boissel J.-P. (2001). A score for predicting risk of death from cardiovascular disease in adults with raised blood pressure, based on individual patient data from randomised controlled trials. BMJ 323, 75–81. doi: 10.1136/bmj.323.7304.75, PMID: 11451781 PMC34541

[ref27] Rand D. G. (2016). Cooperation, fast and slow: Meta-analytic evidence for a theory of social heuristics and self-interested deliberation. Psychol. Sci. 27, 1192–1206. doi: 10.1177/0956797616654455, PMID: 27422875

[ref28] Rand D. G. Greene J. D. Nowak M. A. (2012). Spontaneous giving and calculated greed. Nature 489, 427–430. doi: 10.1038/nature11467, PMID: 22996558

[ref29] Reed A. II Kay A. Finnel S. Aquino K. Levy E. (2016). I don’t want the money, I just want your time: how moral identity overcomes the aversion to giving time to prosocial causes. J. Pers. Soc. Psychol. 110, 435–457. doi: 10.1037/pspp0000058, PMID: 26523999

[ref30] Roxburgh S. (2004). ‘There just aren’t enough hours in the day’: the mental health consequences of time pressure. J. Health Soc. Behav. 45, 115–131. doi: 10.1177/002214650404500201, PMID: 15305755

[ref31] Shah A. K. Mullainathan S. Shafir E. (2012). Some consequences of having too little. Science 338, 682–685. doi: 10.1126/science.1222426, PMID: 23118192

[ref32] Song Y. Chen Q. Ren P. Ma J. Li C. (2024). I’m more prosocial than others: narcissism facilitates prosocial behavior in public situations. Behav. Sci. 14:1200. doi: 10.3390/bs14121200, PMID: 39767341 PMC11672898

[ref33] Spinney J. Millward H. (2010). Time and money: a new look at poverty and the barriers to physical activity in Canada. Soc. Indic. Res. 99, 341–356. doi: 10.1007/s11205-010-9585-8

[ref34] Szollos A. (2009). Toward a psychology of chronic time pressure: conceptual and methodological review. Time Soc. 18, 332–350. doi: 10.1177/0961463X09337847

[ref35] Teoh Y. Y. Hutcherson C. A. (2022). The games we play: prosocial choices under time pressure reflect context-sensitive information priorities. Psychol. Sci. 33, 1541–1556. doi: 10.1177/09567976221094782, PMID: 35994687 PMC9630724

[ref36] Teoh Y. Y. Yao Z. Cunningham W. A. Hutcherson C. A. (2020). Attentional priorities drive effects of time pressure on altruistic choice. Nat. Commun. 11, 1–13. doi: 10.1038/s41467-020-17326-x, PMID: 32669545 PMC7363879

[ref37] Urakawa K. Wang W. Alam M. (2020). Empirical analysis of time poverty and health-related activities in Japan. J. Fam. Econ. Iss. 41, 520–529. doi: 10.1007/s10834-020-09671-2

[ref9001] Wang X. Chen Z. G. Krumhuber E. Chen H. (2021). Money and flexible generosity. British J. Soc. Psychol. 60, 1262–1278. doi: 10.1111/bjso.12450 33604913

[ref38] Wispé L. G. (1972). Positive forms of social behavior: an overview. J. Soc. Issues 28, 1–19. doi: 10.1111/j.1540-4560.1972.tb00029.x

[ref39] Young D. L. Goodie A. S. Hall D. B. Wu E. (2012). Decision making under time pressure, modeled in a prospect theory framework. Organ. Behav. Hum. Decis. Process. 118, 179–188. doi: 10.1016/j.obhdp.2012.03.005, PMID: 22711977 PMC3375992

[ref40] Yuan Y. Sun X. (2024). Can't see the forest for the trees: time poverty influences construal level and the moderating role of autonomous versus controlled motivation. Br. J. Soc. Psychol. 63, 1272–1296. doi: 10.1111/bjso.12730, PMID: 38305091

[ref41] Zhao Q. Ma R. Liu Z. Wang T. Sun X. van J. W.. (2024). Why do we never have enough time? Economic inequality fuels the perception of time poverty by aggravating status anxiety. Br. J. Soc. Psychol. 63, 614–636. doi: 10.1111/bjso.12695, PMID: 37933472

[ref42] Zuzanek J. (2004). Work, leisure, time-pressure and stress. Work and leisure. eds. Haworth J. T. Veal A. J.. (London: Routledge), 137–158.

