# Supplementary material for: Distinct benefit frames generate divergent effects of time scarcity mindset on prosocial behavior
Source: Front Psychol. 2025 Jun 2;16:1601936. doi: 10.3389/fpsyg.2025.1601936 (PMC12168159; doi:10.3389/fpsyg.2025.1601936)
Supplement: Supplementary file 1 [file Supplementary_file_1.docx]

**Supplementary Materials**

to accompany

Distinct Benefit Frames Engender Divergent Effects of Time Scarcity Mindset on Prosocial Behavior

**Table of Contents**

[Measures 2](#_Toc194085912)

[Study 1 2](#_Toc194085913)

[Time scarcity mindset 2](#_Toc194085914)

[Prosocial behavior under the other-benefit frame 2](#_Toc194085915)

[Prosocial behavior under the self-and-other benefit frame 3](#_Toc194085916)

[Control variable: Money scarcity mindset4](#_Toc194085917)

[**Study 2** 5](#_Toc194085918)

[Manipulation and manipulation check of time scarcity mindset, and Attention check items 5](#_Toc194085919)

[Manipulation of benefit frame, measurement of prosocial behavior, and Manipulation check of benefit frame 7](#_Toc194085920)

# Measures

## Study 1

### Time scarcity mindset (Kasser & Sheldon, 2009)

1. My life has been too rushed.

2. I have had plenty of spare time.

3. I have been racing from here to there.

4. I have had enough time to do what I need to do.

5. I have been able to take life at a leisurely pace.

6. There have not been enough minutes in the day.

7. I have had enough time to do the things that are important to me.

8. I have felt like things have been really hectic.

### Prosocial behavior under the other-benefit frame (Adapted from Caprara et al., 2005)

1. If it benefits me personally, I am pleased to help my friends/colleagues in their activities.

2. If it benefits me personally, I share the things that I have with my friends.

3. If it benefits me, I try to help others.

4. For my personal benefit, I am available for volunteer activities to help those who are in need.

5. If it benefits me personally, I am emphatic with those who are in need.

6. If it benefits me personally, I help immediately those who are in need.

7. When it involves my personal interests, I do what I can to help others avoid getting into trouble.

8. For my own career development, I am willing to make my knowledge and abilities available to others.

9. If it benefits me personally, I try to console those who are sad.

10. If it benefits me personally, I easily lend money or other things.

11. If it benefits me personally, I easily put myself in the shoes of those who are in discomfort.

12. If it benefits me personally, I try to be close to and take care of those who are in need.

13. Considering personal benefit, I easily share with friends any good opportunity that comes to me.

14. To ensure companionship when I am in need in the future, I spend time with those friends who feel lonely.

15. I believe that if I help others, they should help me in the future.

### Prosocial behavior under the self-and-other benefit frame (Adapted from Caprara et al., 2005)

1. Even if it does not benefit me personally, I am pleased to help my friends/colleagues in their activities.

2. Even if it does not benefit me personally, I share the things that I have with my friends.

3. Even if it does not benefit me personally, I try to help others.

4. Without considering personal benefit, I am available for volunteer activities to help those who are in need.

5. Even if it does not benefit me personally, I am emphatic with those who are in need.

6. Even if it does not benefit me personally, I help immediately those who are in need.

7. Even when it does not involve my personal interests, I do what I can to help others avoid getting into trouble.

8. Even if it does not benefit my career development, I am willing to make my knowledge and abilities available to others.

9. Even if it does not benefit me personally, I try to console those who are sad.

10. Even if it does not benefit me personally, I easily lend money or other things.

11. Even if it does not benefit me personally, I easily put myself in the shoes of those who are in discomfort.

12. Even if it does not benefit me personally, I try to be close to and take care of those who are in need.

13. Regardless of whether it benefits me personally, I easily share with friends any good opportunity that comes to me.

14. Regardless of whether it benefits me personally, I spend time with those friends who feel lonely.

15. Even if others may not help me in the future, I will still help them.

### Control variable: Money scarcity mindset (Adapted from Kasser & Sheldon, 2009)

1. I have had enough money to buy the things that are important to me.

2. There has not been enough money to go around.

3. I have been able to buy what I want.

4. I have felt like I’m pretty poor.

5. My bank account has been too low.

6. I have had enough money to buy what I need to buy.

7. I have been broke.

8. I have had plenty of spare money.

**Study 2**

### Manipulation and manipulation check of time scarcity mindset, and Attention check items (Adapted from Yuan and Sun, 2024)

Scenario Imagination Task

Please imagine a completely new scenario, which may differ from your current real-life situation. Please imagine this scenario as vividly as possible according to the given instructions, and avoid evaluating your responses based on your current attitudes or behaviors in real life.

Work: Currently, you are employed by Hawei Group, and you have been working there for five years.

Life: You live with your three-person family in the Chunhui community.

We will mark the beginning and end of the task clearly as “Task Begins” and “Task Ends.” Please maintain your new identity when answering the questions within this designated period.

Please remember: the additional participant compensation you receive in this task depends on the choices you make.

**[High time scarcity mindset condition]**

In both your work and personal life, you constantly feel you do not have enough time.

Your work at Hawei Group is extremely busy. Your daily tasks at the company are fully scheduled, and new tasks often emerge before you complete the previous ones. You always feel there is never enough time. You must work quickly and efficiently to meet deadlines. Almost every day, you hurry to your workstation, working from 8:00 a.m. to 6:00 p.m., with only a quick 10-minute lunch break at noon. Frequently, you must work overtime until 9:00 or 10:00 p.m., resulting in over 12 hours of work each day. On weekends, you still have to work overtime due to unfinished tasks, unexpected assignments, or sudden requests from your supervisor.

Aside from your job, you have numerous tasks to manage at home. Before going to work each morning, you need to wake up early to prepare breakfast for your child, who attends primary school, and send them to school. Since your child's school assigns extensive homework that requires parental involvement, you also spend time assisting your child. Additionally, you need to do housework regularly to maintain cleanliness at home. You have no time for leisure activities, no time for socializing with family or friends, and certainly no time for pursuing personal interests or hobbies. Even on weekends, besides catching up on unfinished work tasks, you must accompany your child to extracurricular classes.

In short, your life feels incredibly rushed, with almost no leisure time.

Please vividly imagine and describe your work and life under these conditions (no fewer than 50 words).

**[Low time scarcity mindset condition]**

In both your work and personal life, you feel you have ample time.

Your job at Hawei Group is very relaxed. You always have sufficient time to finish your work tasks and can complete them at your own preferred pace. You arrive at work calmly and punctually each morning, working from 9:00 a.m. to 12:00 p.m., and from 2:30 p.m. to 5:00 p.m., totaling less than six hours of work per day. At midday, you enjoy two and a half hours of leisure time for lunch and rest. You leave work on time every afternoon. Whether it is a weekday or a weekend, you never need to work overtime, leaving you free to arrange your own schedule.

In addition to your job, you have ample time to handle various tasks at home. You can spend plenty of time interacting with and accompanying your child, and you hardly ever worry about household chores. When you wish to relax, you have enough time to read books or watch your favorite TV shows and variety programs. When you wish to exercise, you have ample time to visit the gym. If you decide to go out, you have sufficient time to spend with family or friends. When you want to cultivate a new hobby or interest, you also have plenty of time to participate in related activities.

Overall, you feel very comfortable and have plenty of leisure time.

Please vividly imagine and describe your work and life under these conditions (no fewer than 50 words).

**[Manipulation check of time scarcity mindset]**

Based on the imagined scenario, to what extent do you agree with the following statements? Please use the scale from 1 (Strongly Disagree) to 7 (Strongly Agree). A higher score indicates greater agreement. Living in the imagined scenario above,

1. I feel pressed for time.

2. My time is always insufficient.

3. I often feel rushed.

**[Attention check items]**

Which of the following accurately describes the scenario you have just imagined?

1. I do not need to work overtime and have no child.

2. I need to work overtime and have no child.

3. I do not need to work overtime and have a child.

4. I need to work overtime and have a child.

### Manipulation of benefit frame, measurement of prosocial behavior, and Manipulation check of benefit frame

Your community, Chunhui Community, is organizing a fundraising campaign aimed at assisting families in need. You have received a notification informing you that you have a limited time to participate in this donation campaign. This fundraising activity will directly impact disadvantaged groups helped by this event. You can choose either to donate funds or to not participate.

**[Other-benefit frame]**

You may choose to contribute funds to this campaign. The community implements anonymous donations, and your donation amount will not influence any future arrangements or activities. Therefore, you will not receive any personal benefit from your donation. Your contribution is purely altruistic, intended solely to help others in need, and will not yield any economic or social returns for you.

The system has allocated 1,000,000 tokens to you. These 1,000,000 tokens can be converted into an additional participation reward of 1 CNY. According to the rules of this task (i.e., donations are anonymous and will not bring any personal benefit), if you donate X (ten-thousand) tokens, your additional participation reward will be calculated as: (100 - X) / 200 CNY.

For example, if you donate 100,000 tokens (10 units of ten-thousand tokens), your additional reward will be (100 - 10) / 100 = 0.9 CNY.

**[Prosocial behavior under the other-benefit frame]**

How many tokens will you donate?

Slider: from 0 to 100 (units of ten-thousand tokens)

**[Self-and-other benefit frame]**

You may choose to contribute funds to this campaign. Although the community implements anonymous donations, the community will organize various charity activities tailored to groups based on their donation amounts. Thus, through this activity, in addition to helping others, you can also gain personal benefits such as improving personal skills, expanding your social network, and potentially gaining career development opportunities or additional educational resources for your child. In other words, your contribution benefits others as well as positively impacts your future.

To facilitate this donation campaign, the system has allocated 1,000,000 tokens to you. These 1,000,000 tokens can be converted into an additional participation reward of 1 CNY. According to the rules of this task (i.e., through this activity, besides helping others, you can gain personal benefits such as skill enhancement, networking opportunities, potential career advancement, or educational resources for your child), if you donate X (ten-thousand) tokens, the system will calculate your additional personal return as 1.5X (ten-thousand) tokens. Consequently, your final additional reward will be: (100 - X + 1.5X) / 200 CNY.

**[Prosocial behavior under the self-and-other benefit frame]**

How many tokens will you donate?

Slider: from 0 to 100 (units of ten-thousand tokens)

**[Manipulation check of benefit frame]**

Based on the scenario described above, to what extent do you agree with the following statements? Please rate each item from 1 (*Strongly Disagree*) to 7 (*Strongly Agree*):

1. Donating benefits me personally.

2. Donating not only helps others but also benefits me.

3. Donating is solely for helping others and provides no personal benefit to me.
